# Digitally enhanced recovery: Investigating the use of digital self-tracking for monitoring leisure time physical activity of cardiovascular disease (CVD) patients undergoing cardiac rehabilitation

**DOI:** 10.1371/journal.pone.0186261

**Published:** 2017-10-11

**Authors:** Jürgen Vogel, Andreas Auinger, René Riedl, Harald Kindermann, Markus Helfert, Helmuth Ocenasek

**Affiliations:** 1 School of Management, University of Applied Sciences Upper Austria, Steyr, Austria; 2 CARDIOMED Outpatient Cardiac Rehabilitation Centre, Linz, Austria; 3 Department of Business Informatics-Information Engineering, Johannes Kepler University, Linz, Austria; 4 School of Computing, Faculty of Engineering and Computing, Dublin City University, Dublin, Ireland; Worcester Polytechnic Institute, UNITED STATES

## Abstract

Research has shown that physical activity is essential in the prevention and treatment of chronic diseases like cardiovascular disease (CVD). Smart wearables (e.g., smartwatches) are increasingly used to foster and monitor human behaviour, including physical activity. However, despite this increased usage, little evidence is available on the effects of smart wearables in behaviour change. The little research which is available typically focuses on the behaviour of healthy individuals rather than patients. In this study, we investigate the effects of using smart wearables by patients undergoing cardiac rehabilitation. A field experiment involving 29 patients was designed and participants were either assigned to the study group (N = 13 patients who finished the study and used a self-tracking device) or the control group (N = 16 patients who finished the study and did not use a device). For both groups data about physiological performance during cardiac stress test was collected at the beginning (baseline), in the middle (in week 6, at the end of the rehabilitation in the organized rehabilitation setting), and at the end of the study (after 12 weeks, at the end of the rehabilitation, including the organized rehabilitation plus another 6 weeks of self-organized rehabilitation). Comparing the physiological performance of both groups, the data showed significant differences. The participants in the study group not only maintained the same performance level as during the midterm examination in week 6, they improved performance even further during the six weeks that followed. The results presented in this paper provide evidence for positive effects of digital self-tracking by patients undergoing cardiac rehabilitation on performance of the cardiovascular system. In this way, our study provides novel insight about the effects of the use of smart wearables by CVD patients. Our findings have implications for the design of self-management approaches in a patient rehabilitation setting. In essence, the use of smart wearables can prolong the success of the rehabilitation outside of the organized rehabilitation setting.

## Introduction

A healthy lifestyle, which is a key determinant in the prevention and treatment of chronic diseases, involves regular physical activity [1]. Evidence indicates that physical activity is a major determinant of chronic diseases, in particular of cardiovascular disease (CVD) [2–6]. Less activity than 5,000 steps per day, for example, is associated with unfavourable indicators of cardiometabolic risk [7]. Furthermore, sedentary behaviour is known to influence the risk for chronic conditions such as CVD [8]. Today’s living environments (e.g. transport facilities, workplace designs, and electronic communication), however, foster diminished physical activity. Thus, if compared to the past, the way modern humans move and sit in everyday life has changed fundamentally in the last few decades [6,8].

Concurrently, in recent years there has been an increasing interest in self-tracking through computer technology to improve health and medical conditions. Specifically, the increased ability of individuals to perform self-monitoring through smartphone apps and web services contributed to this development. In research areas such as obesity studies (self-tracking of weight loss), psychology (collecting mood data), or diabetes care, research has already established positive effects [9,10]. However, current research on the effects of smart wearables (e.g., activity trackers) has primarily focused on healthy individuals rather than patients.

In outpatient cardiac rehabilitation, besides scheduled physical exercise sessions in a rehabilitation environment, leisure time physical activity (LTPA) has been shown to positively affect health conditions [11–13]. Thus, to make changes towards a healthy lifestyle, patients should increase their activities and reduce time spent in a seated position. Giving them the possibility to digitally track their behaviour (e.g., daily steps) may increase awareness of LTPA including sedentary behaviour, and this, in turn, may lead to more LTPA, thereby improving the cardiovascular health status [5,14,15].

Consumer wearable devices for activity tracking promise an enhancement of recovery in cardiac and pulmonary rehabilitation [16]. Specifically, such devices enable the monitoring of activities during the recovery process in real life (e.g., at home or in community settings), allow medical professionals to collect data from individuals (e.g., to monitor whether patients are following their recommendations during rehabilitation [17]), and provide a new way to determine improvement or deterioration of health [18–20]. Such devices also provide support to outpatient cardiac rehabilitation services and offer a cost-effective and feasible alternative to hospital-based rehabilitation programmes and services [21,22]. Thus, use of consumer wearable devices may improve the allocation of healthcare resources and increase efficiency and quality of healthcare systems [23]. Altogether, health tracking devices can be used to address the behavioural risk factors of CVD by encouraging an increase in LTPA [24,25], a fact that holds particularly true for the prevention of coronary heart disease [26].

However, current studies on physical activity are mostly based on questionnaires and subjective self-reporting by healthy individuals, and tend to focus on specific activities and less on routine daily activities of light intensity [1,4]. Also, the usage of activity monitors to measure physical activity in clinical contexts is limited [27]. Napolitano et al. write that an “objective measurement of physical activity behaviour is a critical missing component in physical activity clinical trials” (p. 521 of [28]). Although the use of consumer wearable devices is promising for healthcare, it is still in the early stages of development and has rarely been approved for medical use by real patients [29]. Also, there are only a few studies providing insight about the use of wristband activity trackers for monitoring and improving health outcomes among the elderly [30]. Thus, a research gap exists on the effects of the use of consumer wearable devices by chronic disease patients for self-management on health outcomes [16].

To the best of our knowledge, no controlled peer-reviewed studies that investigate the monitoring of patients’ LTPA by wearing an activity tracker during and after outpatient cardiac rehabilitation have been published. It follows that insights about a tracker’s role and its potential effects on the development of patients’ physical activity and health is limited. Wrist-worn activity trackers given to patients should help them to record their LTPA and support them in monitoring these activities. This should lead to patients having a greater awareness of their own physical activity behaviour. Therefore, in the present study we investigate the following hypothesis:

*Patients undergoing cardiac rehabilitation who objectively record leisure time physical activity by using a digital activity tracker show higher improvement of the cardiovascular system after 12 weeks than patients undergoing cardiac rehabilitation who do not objectively record leisure time physical activity by using a digital activity tracker*.

The remainder of this paper is structured as follows: in the first section, we provide background knowledge on physical activity and health, including a more specific discussion of CVD and physical activity during rehabilitation. Afterwards, we review literature on smart wearables and their application in health monitoring. Both sections constitute the conceptual basis of the present study and provide insight into the rationale underlying the hypothesis that we tested in a field study. What follows then is a description of our research approach, including a description of our study population and the methods. Afterwards, we present our results, grouped into the descriptive statistics of activity data and the hypothesis testing. We close this paper with a discussion of the findings, including an outline of the study’s limitation and a concluding comment.

### Physical activity and health

The intensity level of physical activity is commonly referred to as light, moderate, and vigorous [31]. On an absolute scale, moderate intensity refers to activity that is performed 3.0–5.9 times and vigorous intensity 6.0 or more times the intensity of rest [3], depending on people’s skill level and body composition [4]. The most recent evidence-based recommendations for adults (aged 18 to 65) on physical activity and public health call for at least 30 minutes of moderate-intensity physical activity five days a week, 20 minutes of vigorous-intensity physical activity three days a week or an equivalent combination of moderate to vigorous physical activity (MVPA). Exceeding the minimum recommended amounts has been shown to reduce the risk for chronic diseases [1,4]. If adults are unable to follow up recommendations due to health conditions, they should, nevertheless, be as physically active as possible. They can still benefit from amounts below the recommended threshold, particularly if they are habitually inactive [6]. However, although findings show the beneficial effects of MVPA [15], these activities decline with increasing age [32] and especially older adults tend to spend the majority of their time engaged in sedentary or light physical activity. Reducing total sedentary time and replacing it with light physical activity or short periods of standing, has beneficial effects on health, even in physically active adults [1,12]. Counted steps explain the majority of the daily variability in time spent on MVPA [7]. Reaching a daily step-count of about 7,000 steps or more is classified as being physically active [1].

CVD is the single largest cause of death worldwide [33]. Although rates of CVD death have declined, still almost a third of all deaths are attributed to CVD (30.8%) [2]. Physical activity not only plays a decisive role in staying healthy and preventing chronic diseases, but is also a key factor in the treatment of diseases, particularly CVD [11,34]. Cardiac rehabilitation involves a variety of therapies by an interdisciplinary team and consists of exercises, education, and psychological support. Its general objectives are to regain fitness and health, thereby enhancing quality of life and reducing the likelihood of dying from heart disease [35–37] or to “prevent subsequent coronary events, subsequent hospitalization, and death from cardiac causes through a program of prescribed exercise and interventions designed to modify coronary risk factors” (p. 892 of [38]). Generally, it “is indicated for patients who have received a diagnosis of acute myocardial infarction, those who have undergone coronary revascularization, and those with chronic stable angina” [38].

In Austria, the country in which the empirical study reported in this paper was conducted, the follow-up treatment after early mobilization or a hospital stay after an acute cardiac event is referred to as phase II rehabilitation with a duration of 6 weeks. It is either hospital/centre-based or outpatient/home-based, both producing similar favourable outcomes for patients who adhere to corresponding guidelines [39,40]. During phase II rehabilitation endurance and resistance training takes place three times a week (up to 50 minutes) under supervision of a rehabilitation physician and a sports scientist (exercise physiologist). All programmes follow the Austrian Guidelines of Outpatient Cardiac Rehabilitation, which have been endorsed by the Austrian Society of Cardiology [41,42]. Patients’ exercise capacity usually improves through rehabilitation by 20 watts (W; watt as in the unit of power), and rehabilitation leads to improvement in cardiovascular risk factors (e.g., systolic blood pressure, BMI, or psycho-cardiac parameters) [43]. Moreover, the average MVPA increases significantly during a 12-week home-based cardiac rehabilitation programme [37].

### Smart wearables and monitoring in healthcare

#### Consumer wearable devices and activity trackers

Wearable devices, which can be defined as “mobile electronic devices that can be unobtrusively embedded in the user’s outfit” (p. 232 of [44]), are able to recognize the user’s specific situations due to context sensitivity [44]. Their main purpose is to collect and analyse data on the individual’s state and behaviour [45]. In recent years, wearable devices for tracking purposes have developed at a stunning pace due to cheap computer storage and rich user interfaces [46,47].

Activity trackers support people trying to improve their health, physical performance, or overall wellness by monitoring their activities and activity-related metrics (e.g., kcals burned) in their everyday lives [48]. They are mostly designed as bracelets with a graphic display or vibration for user-device interaction [49]. In general, studies investigating activity trackers show high measurement validity and inter-device reliability of step-counts, but lower validity for energy expenditure and sleep assessment [48]. Although most wearable devices are accurate for tracking purposes, in some cases a more than 20% deviation to actual step-counts was found [50]. Causes of poor reliability can be related to the inaccuracy of sensors, the failure of software algorithms or the users themselves who try to intentionally cheat the recognition system (e.g., by shaking the device) to appear more active [17].

#### Wearables and healthcare

Wearables enable patients to take an active role in their healthcare and prevention of diseases by giving feedback on the relation between physiological parameters and activities [45]. While tracking ourselves we are collecting detailed data about our body, which can be easily kept for records [51]. Therefore, wearable technology is becoming integrated in our everyday lives [52] and “[t]he line between consumer health wearables and medical devices begins to blur” (p. 1 of [29]). Generated data gives individuals access to personal analytics and could help in managing medical risk factors. Wearables could monitor patients’ progress in an easy and comfortable way and “could provide a platform for at-home management of long-term chronic conditions” (p. 3 of [29]). New technologies and data about behaviour enable doctors to look after patients during their LTPA, regardless of geographical distances or boundaries [53]. Monitoring someone’s physical activity can increase overall activity significantly [54]. It has been shown that outpatient adults using pedometers significantly increased their own step-count or activity per day [55]. In general, the technological development of self-monitoring systems has been advancing faster than the rate at which human society is able to understand its consequences. The generated data gives a powerful and intimate insight into ourselves, including both human physiology and behaviour [56].

#### Measuring methods

Basically, methods for measuring physical activity are divided into two groups: self-reports and objective measures (e.g., motion sensors such as accelerometers) [6,11]. Self-reports like questionnaires, have been the most commonly used method for assessing physical activity. However, self-reports constitute subjective assessment, and hence a number of measurement biases may occur, such as memory distortion or social desirability bias [14,28,57,58]. Discrepancies between self-report measures of physical activity and those directly measured are reported [59]. It follows that questionnaires for assessments of physical activity are limited regarding reliability and validity [60,61].

While adults typically subjectively overestimate their physical activity or its intensity in self-reports, accelerometers provide approximately true values, [14,57]. Moreover, older adults face difficulties in recalling the intensity, frequency, and duration of their activity [12]. Therefore, at present, accelerometers seem to be one of the most reliable and relatively affordable alternatives to gather detailed information about physical activity and its intensity levels. They are defined as “devices that measure body movements in terms of acceleration, which can then be used to estimate the intensity of PA [physical activity] over time” (p. 490 of [62]). Compared to pedometers, which only measure the presence of an actual movement, accelerometers measure the magnitude of force generated by movement, which is referred to as acceleration. Activity trackers also use this to estimate energy expenditure (e.g., in the form of Kcal burned) [27].

Technically, the generated data is first recorded by an internal memory and then transferred to computers or Internet servers. The total daily activity, the time spent at different intensity levels, changes in activity patterns, and the characterisation of sedentary behaviour are widely used measures. To obtain most reliable and valid results from a clinical research perspective, the use of a triaxial accelerometer worn on a participant’s non-dominant wrist is recommended. To capture both weekdays and weekends the minimum period of wear should be 7 days. Although, obviously, most detailed outcomes tend to be achieved by wearing activity trackers 24 hours a day, a minimum of 12 hours’ wear time per day generally results in accurate estimates of daily physical activity [27].

Basically, the perception about the feasibility and utility of wristband activity trackers is positive among adults aged 60 or older [30]. The acceptability of apps based on motivational frames of behavioural science theory which should help to increase MVPA and reduce sedentary time is also confirmed for adults aged 45 or older who were not familiar with the technology and insufficiently physically active [63]. Furthermore, devices attached to the wrist seem to be the most readily accepted manner for monitoring physical activity by older adults [64].

#### Behaviour change

Although we know that lifestyle is essential for the prevention and treatment of chronic diseases like CVD, it is challenging for people to change their lifestyle. Reasons for this may be a person’s socio-economic status (e.g., low educational level), social isolation (e.g., living alone), stress, negative emotions (e.g., depression), or that recommendations for physical activity could be misunderstood [6].

Using wearables for healthcare purposes has psychological effects on the patient [18] and behaviour or cognitive behaviour change can support the assistance of patients [65]. Computer-based solutions can effectively cause behavioural change to help prevent chronic diseases such as CVD [66]. Activity trackers may act as an interactive behaviour change tool as they can provide immediate feedback to the user. They show the daily progress of physical activity and enable sharing and comparing data with other users, both of which could be a motivation to increase overall physical activity. Activity trackers also use a variety of potentially effective behaviour change techniques known from typical clinical interventions. They include, for example, goal-setting, showing differences between current and goal behaviour, visual cues, self-monitoring, or motivating feedback [67]. Against this background, activity trackers and their applications seem to hold significant potential for use in rehabilitation settings. In general, mobile technology interventions in healthcare are effective and already show benefits in different domains, such as increased physical activity in the short term and improved cardiovascular risk profiles [68].

## Field experiment

A field experiment with patients undergoing rehabilitation was performed at the outpatient cardiac rehabilitation centre Cardiomed in Linz, Austria in cooperation with the School of Management of the University of Applied Sciences Upper Austria and Dublin City University. The study was approved by the responsible Management Board of the University of Applied Sciences Upper Austria and the Dublin City University Research Ethics Committee (REC Reference: DCUREC/2016/011). Both confirmed that the study was conducted under mutual consideration of scientific and ethical aspects. (Written) Informed consent was given by all participants.

### Method

Randomly selected male patients who attended outpatient cardiac rehabilitation phase II (follow-up treatment after early mobilization or hospital stay after an acute cardiac event) were divided into two groups by a standard software-based random sample generator. One group of patients, referred to as ‘study group’ (N = 19), was provided with activity trackers for a duration of 12 weeks and asked to perform a supplementary examination at the end of their observation period. Another group of patients, referred to as ‘control group’ (N = 17), was asked to undergo a supplementary examination 12 weeks after commencement of their rehabilitation without any further intervention (no activity tracking). To compare improvement of different parameters (e.g., performance of cardiovascular system) between the study and control group, results based on three different examinations (*t*1, *t*2, *t*3) were analysed.

The total duration of the field experiment was 29 weeks over a period of 8 calendar months (from October 2015 to May 2016). At the outpatient cardiac rehabilitation centre usually a group consisting of 12 patients (mainly men) starts rehabilitation every two weeks. Participants were recruited from these routine rehabilitation groups on the day of their baseline examination. The groups for the study were designed to avoid an overlap of study and control group participants in the same routine rehabilitation group to ensure that participants of different groups could not influence each other. The baseline examination took place at the beginning (in week 1) and the midterm examination at the end (in week 6) of the rehabilitation programme. A supplementary examination, which was only performed as a consequence of this field experiment, took place after 12 weeks (in week 13). The observation period (period for tracking and monitoring of physical activity) was therefore 12 weeks per participant.

Our step-by-step laboratory protocol is available in protocols.io: https://dx.doi.org/10.17504/protocols.io.jhacj2e. [PROTOCOL DOI]

#### Participants

Physical activity levels may vary depending on gender [37]. Further, patients at the outpatient cardiac rehabilitation centre are predominantly male. To avoid the risk of skewed results influenced by gender differences, and to enable recruitment of an adequate number of participants within a limited time frame, only male participants were included in the study. Additional inclusion criteria were that participants were aged 40–80 and had been diagnosed with CVD (ST-elevation myocardial infarction (STEMI), non-ST-elevation myocardial infarction (NSTEMI), percutaneous coronary intervention (PCI) or coronary revascularisation) within the previous 3 months. Eligible participants had been clinically stable for at least two weeks before the start of the study and were able to participate in the rehabilitation programme, including the performance of the recommended physical activity. They had to give informed consent, and had a personal computer with access to the Internet at home. Patients were excluded if they were suffering from Parkinson's disease or other medical conditions such as uncontrolled atrial or ventricular dysrhythmias, or if they were unable to participate in MVPA due to physical limitations.

All participants received detailed information in the form of an introductory face-to-face conversation (duration approximately 30 minutes) conducted by a subset of the authors of this paper; the conversation was about the purpose and procedure of the study, benefits and risks to the participants, effects on medication and lifestyle, expected symptoms, side effects or injuries, information about premature termination of participation, use of data and results, costs of participation, reimbursement of costs and remuneration, and contact details for further inquiries. Additionally, all information was provided to participants in written form in easily understandable language. The consent form was signed by the patient and the responsible author only after all patient questions were answered satisfactorily; hence the patient completely understood the way in which the study was to be conducted, was aware of his rights as a participant, was willing to consent to participation and confirmed that he had had enough time to think about whether he wished to participate in the study or not.

All participants attended routine cardiac rehabilitation during the first six weeks of the study with heart rate controlled moderate to vigorous physical activity, nutritional education, and psychological support. Exercises were partly supervised at the rehabilitation centre and partly conducted at home. The recommendation and duration of the patients’ medication were not affected by this field experiment and therefore remained unchanged. Further, there was no additional medication for reasons related to this study. After the examination six weeks from start participants got medical recommendations for their future lifestyle (including physical activity). For the following six weeks (until the supplementary examination in week 13) the study group participants continued to wear the activity tracker. Thus, for participants in the control group, as in their first six weeks, there was no intervention until the supplementary examination.

All participants were Austrian citizens. Nineteen male cardiac patients undergoing rehabilitation were recruited to participate in the study group. Six of them were unable to complete rehabilitation for medical reasons (not related to the field experiment) at different times throughout the study and had to terminate their participation. Therefore, 13 participants of this group ultimately finished the study and attended all examinations. Seventeen male patients were recruited to participate in the control group, one of whom was unable to complete rehabilitation for medical reasons (unrelated to the field experiment). Therefore, 16 participants of this group ultimately finished the study and attended all examinations. The total number of participants recruited for both groups combined was 36; the total number of participants finishing the study was 29. The mean age on the day of rehabilitation commencement (initial examination) was 61.69 (SD = 8.62; max = 75; min = 44) for the study group and 63.75 (SD = 10.32; max = 77; min = 44) for the control group. There was no statistically significant difference in age between the study and control groups (*p* = .584).

#### Examinations

At the beginning (*t*1), in week 6 (*t*2), and after 12 weeks (*t*3) an examination of the participants’ physiological performance (including lactate measurement) took place at the rehabilitation centre. All measures were performed by a medical doctor or a trained exercise physiologist (under supervision of a medical doctor, to deal with any emergencies). A graded exercise test (cardiac stress test) was conducted on an electronically braked cycle ergometer. The exercise test enables the determination of a participant’s maximum exercise capacity [69]. It is a standard diagnostic process in cardiology and follows the latest Practice Guidelines for Exercise Testing [70] which provide recommendations about the procedure of testing, the valuation of measured parameters and possible influences. Furthermore, they give an overview of interruption and termination criteria. In our study, individual ramp protocols were used according to the latest national guidelines [42,70] and international recommendations [69,71,72]. To ensure comparability of measures of *t*1, *t*2 and *t*3, these were kept identical for all participants during the different examinations. Heart rate was monitored continuously by a conventional 12 lead ECG with ten electrodes. Blood pressure was measured manually with a standard medical measuring device. Participants performed maximum capacity exercise testing until exhaustion or until individual ECG termination criteria were reached. In general, we chose testing protocols lasting 8–12 minutes to the peak of exercise depending on the participant’s physical status and capacity [70,71]. For the test, after an initial warm-up phase with unloaded pedalling, the work rate during progressive uninterrupted exercise is regularly increased after an adequate time interval in each level until the participant’s maximum work rate (power given in W) is reached [69,71]. Therefore, our values and parameters used for comparison of the participants’ physiological performance between examination *t*1, *t*2 and *t*3 and between the study and the control groups were the maximum power given in W achieved by the participants during the cardiac stress test and their calculated relative performance as a percentage (representing their actual performance during the cardiac stress test in relation to their individual target values) [70].

#### Monitoring physical activity

The tracking and monitoring of study group participants’ leisure time activity was performed using the Polar Loop activity tracker which is a common consumer smart wearable device. It is a bracelet-shaped, wrist-worn 3D accelerometer (triaxial accelerometer) with a storage capacity of at least 7 days. This device only has basic features and allows for easy navigation through the display via a single button.

Activity trackers were distributed to participants (free of charge) on the day of *t*1 and returned on the day of *t*3. In order to synchronize data, devices had to be connected to a USB port of a computer, where data was visualised. Participants were asked to wear the device on their non-dominant wrist, if possible, 24 hours a day and seven days a week, to regularly charge the (rechargeable) battery via computer (USB) or charging unit, and to regularly synchronize the automatically collected activity data by using the provided software. Important information about display views, features and functions, memory capacity, charging and operating time, communication and technical specifications of the device, as well as system requirements for the software and web service, was provided to the participants during the introductory conversation. Furthermore, and to ensure that the device was comfortable to wear, the bracelet size was individually customised to the wrist size of all participants. Additionally, all participants received a copy of the user manual.

The device is based on non-invasive sensors and can be classified as a mobile, on-body system. Basically, it is a standalone system in which the collection (embedded sensors), analysis (locally), and visualisation (on display) of data is done by the device itself. Because of the limited storage capacity of the device, data needs to be synchronised with a computer about once a week to enable ongoing tracking. The switch between various modes (display views) is performed via a single touch button. A numeric and graphic display consisting of 85 LED lights in a 5 x 17 matrix gives feedback in the form of numbers and visualisations. All features of the device are based on acceleration measurement. Therefore, the acceleration signal counts and filters body movement, which is then classified into different intensity levels. The model uses intensity levels (and thresholds) based on scientifically approved recommendations [1,3,4]: *non-wear* (device not worn), *sleep/rest* (lying down), *sitting* (including other passive behaviour), *low intensity activity* (light), *medium intensity activity* (moderate), and *high intensity activity* (vigorous). Based on the different intensity levels and body parameters like age, gender, or weight, values are further transferred into step-counts and energy expenditure in the form of burned kilocalories (kcal). Feedback about daily activity is given textually (steps, kcal) and via progress bar shown on the display of the device. After one hour of sedentary behaviour a so-called ‘inactivity alert’ reminds the user to break up sitting time by showing the text “It’s time to move” [73].

Data was automatically generated by participants while wearing the activity tracker. For synchronizing data with a computer, participants had to download and install the free software *Polar FlowSync* on their own PC or laptop. As some of the elderly people were not quite familiar with this kind of process, it was explained to them in detail. Additionally, they received a copy of an installation guide with step-by-step instructions (including screenshots) created by the authors. Once the installation was completed and the device connected to the computer via Polar’s custom USB cable the *Polar Flow web service* opened automatically in the default browser. Nineteen e-mail addresses (Gmail accounts) and Polar accounts (necessary for using the free web service) were prepared by the authors in advance. Activity trackers were set up, updated to the latest software version, and connected to a Polar account. The study reference number (which was also used as the name for the participants’ Polar Account), date of birth, gender, weight, and height of every participant were also added to the accounts in advance. Additionally, the privacy preferences of the web service were set to the most private level. Participants received personal login data to access a pre-set account immediately after they had agreed to participate in the study. Accounts were connected to a special coaching account held by the authors. This enabled the authors to precisely monitor all synchronised activity data of participants. Participants were informed that only the authors and medical staff at the rehabilitation centre have access to their activity data and that all personal information such as full name and date of birth is subject to medical confidentiality. For statistical purposes and analysis all data was anonymised to the study reference number so that it could not be traced to an individual based on published results of the study.

Participants were advised to try to raise their awareness about their own physical activity behaviour, to set realistic and achievable personal goals (such as total daily steps, less overall time spent sitting, or just being more active than the day before) and not to focus too much on the goals suggested by the activity tracker itself, which are generally based on recommendations for healthy individuals rather than cardiovascular patients (goals could not be customised according to individual characteristics of the patients’ disease or diagnosis).

Every three days the authors checked whether participants were synchronising their activity data regularly. If anyone’s data was missing for more than five days, participants were contacted (personally, by phone, or per e-mail), received a reminder to synchronise data, or were asked whether there were any problems (e.g., with synchronisation or use of the device).

## Results

All statistical analyses were performed with IBM SPSS Statistics version 20. The data behind visualizations in this section are available in S1 and S2 Data.

### Descriptive statistics of activity data

Over a total period of five calendar months (18 calendar weeks) activity data was collected from participants of the study group. Only valid days with at least twelve hours of wear time per day were used for data analysis. For statistical analysis, only valid days of participants who finished the study and attended all examinations were used. In total, 1,059 valid days were tracked by 13 participants with a mean number of 81.46 days per participant (SD = 5.33; max = 86; min = 65). Only weeks with a total of seven valid days were declared as valid weeks. In total, 163 weeks (including weeks 1 and 12) were tracked, of which 141 weeks were declared as valid. The mean number of valid weeks was 10.85 weeks per participant (SD = 1.70; max = 12; min = 6).

Activity data in this study was collected as number of steps, kcal burned, sedentary behaviour, total activity time (light + moderate + vigorous intensity) and physical activity of light intensity in hours (h), and MVPA in min. To develop better insight into an actual activity profile of CVD patients some results are presented as line charts in this article.

**Fig 1:**
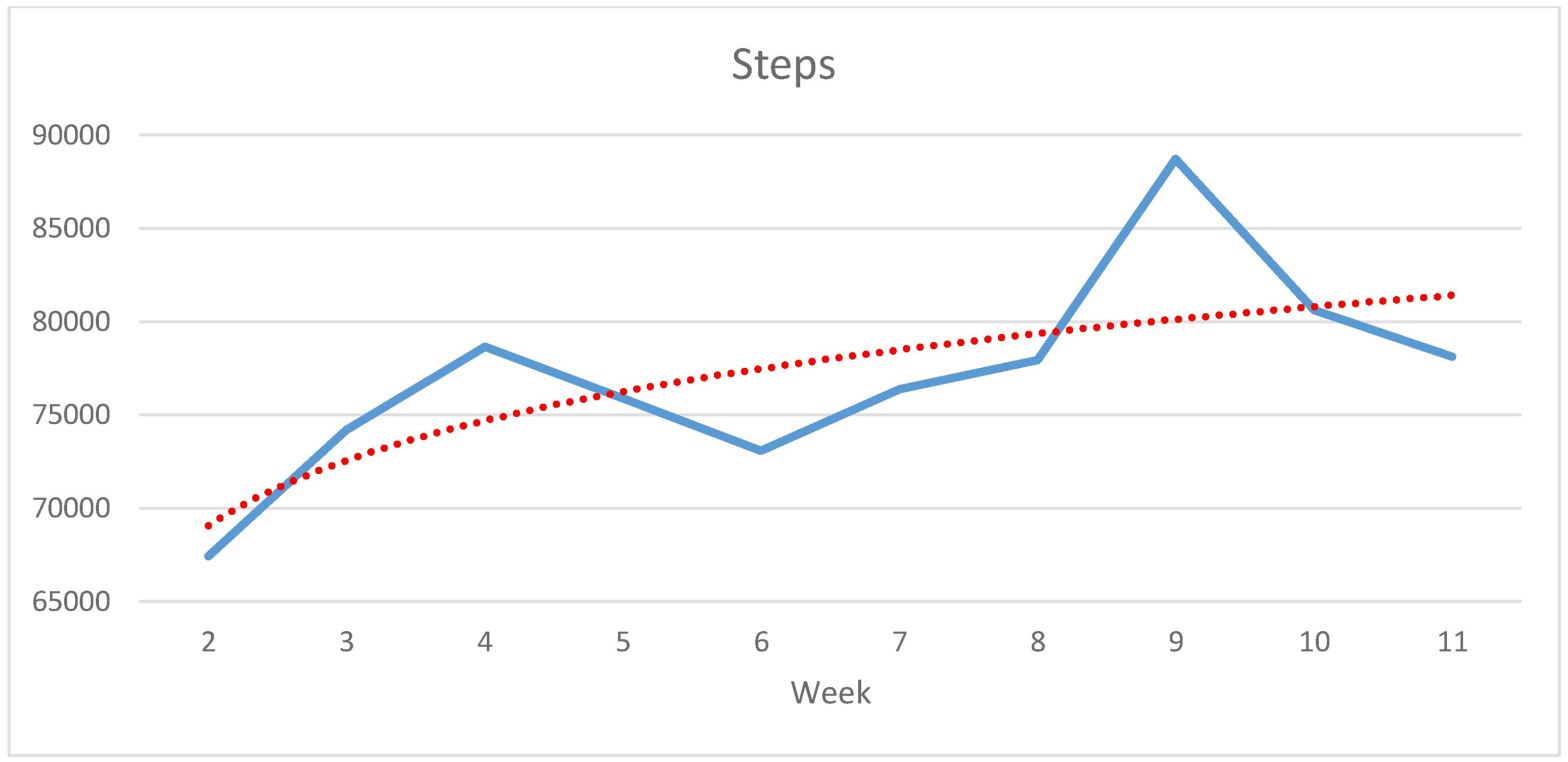
Development of average weekly steps from weeks 2 to 11 (N = 13, study group).

**Fig 2:**
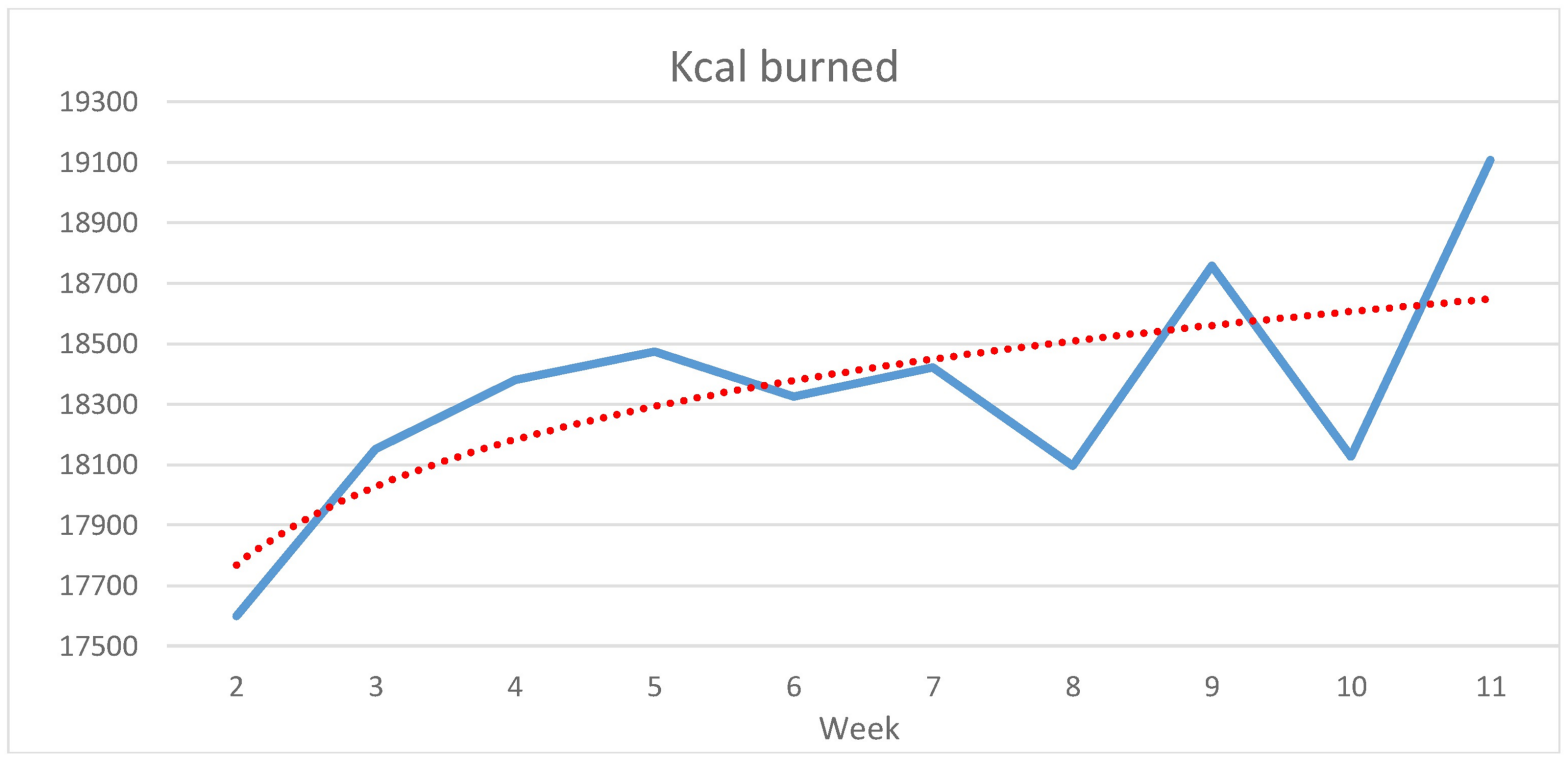
Development of average weekly burned kcals from weeks 2 to 11 (N = 13, study group).

The participants’ average number of weekly steps (Fig 1) increases in the middle of the first and second half of the study, fluctuates somewhat in between, and reaches its peak in week 9. Compared to the beginning of rehabilitation (in week 2) the weekly number of steps increases by almost 21,300 (+31.59%). Participants’ average of weekly burned kcal (Fig 2) increases almost steadily over the first five weeks and then fluctuates after the end of the routine rehabilitation programme in week 6. The difference between week 2 and the peak in week 11 is 1,509 kcals (+8.57%). According to the regression lines, both steps and overall energy consumption climbed steadily over the entire observation period.

**Fig 3:**
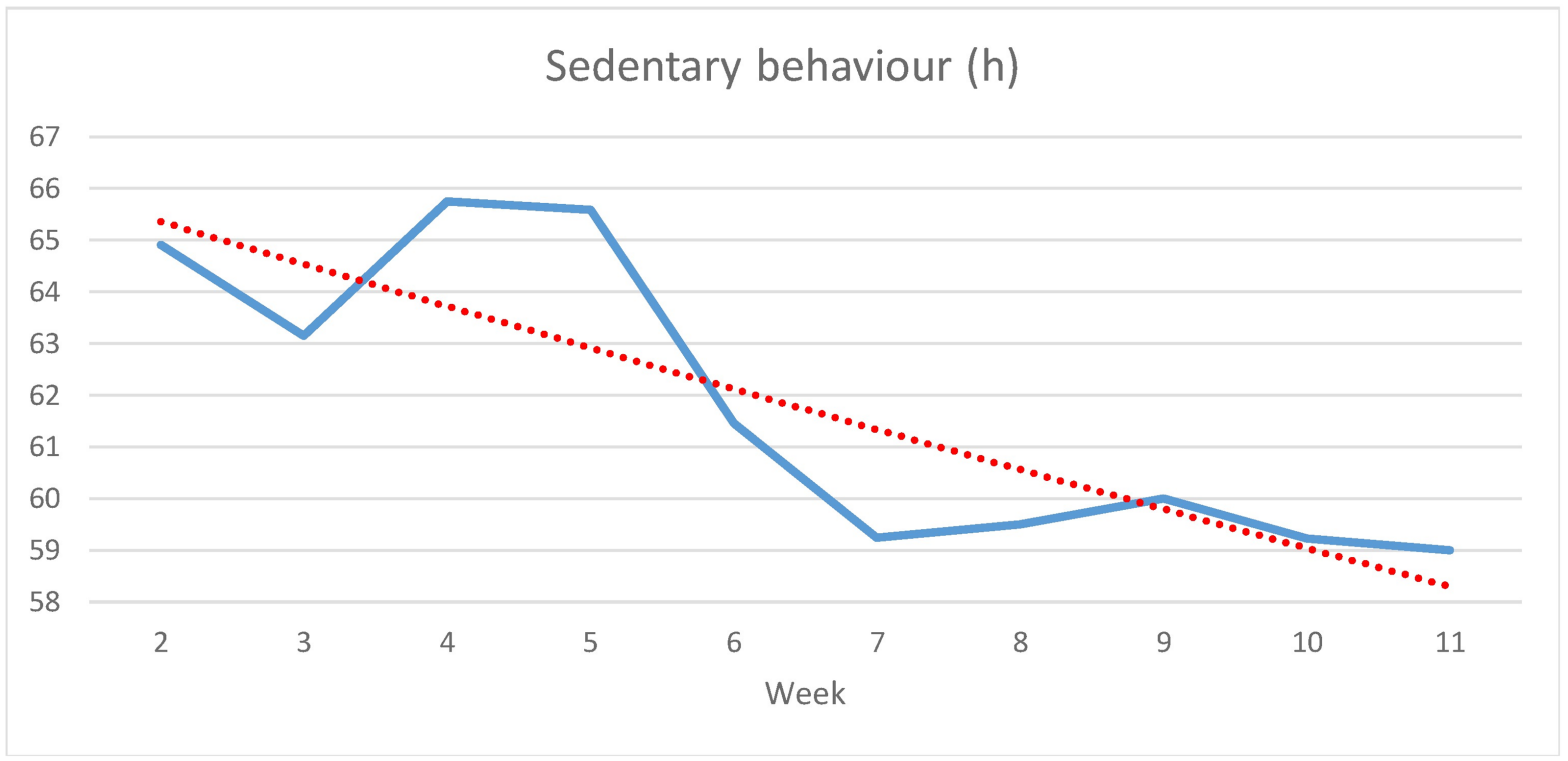
Development of average weekly sedentary behaviour in h from weeks 2 to 11 (N = 13, study group).

Fig 3 shows participants’ average time of sedentary behaviour in h. It increases somewhat from weeks 2 to 3, time edges up again to its peak in week 4 and then decreases until week 7. After that it fluctuates slightly but stays nearly steady through week 11. At the end of the entire period from weeks 2 to 11 participants spend almost 6 h less sitting compared to the beginning (-9.09%).

**Fig 4:**
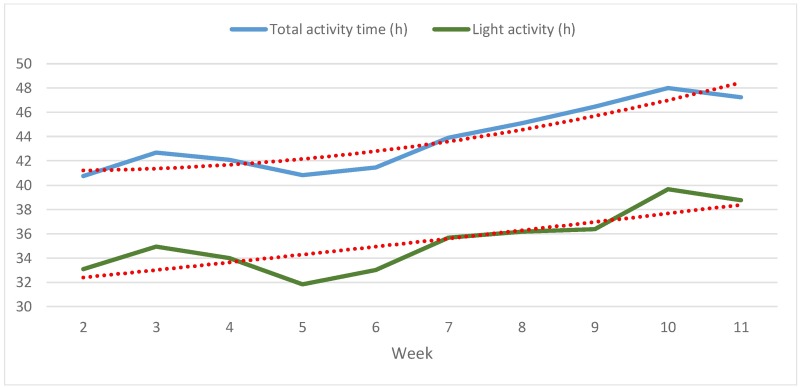
Development of average weekly total and light activity time in h from weeks 2 to 11 (N = 13, study group).

Fig 4 shows participants’ weekly average total activity time (light, moderate, and vigorous intensity combined) and time of light activity in h separately. After increasing from weeks 2 to 3, total activity time edges down again until week 5. Afterwards, it rises by 7.2 h (17.65%) from 40.8 up to 48.0 h in week 10. Time of light activity increases from weeks 2 to 3, decreases until week 5 and then climbs again until week 7. Afterwards it stays almost flat for 2 weeks and then reaches a peak in week 10. Regression lines show a steady climb of total activity time throughout the study and an overall improvement of activity of light intensity by almost 6 hours (+21.21%) from about 34 h in week 2 to almost 40 h at the end of the observation period.

**Fig 5:**
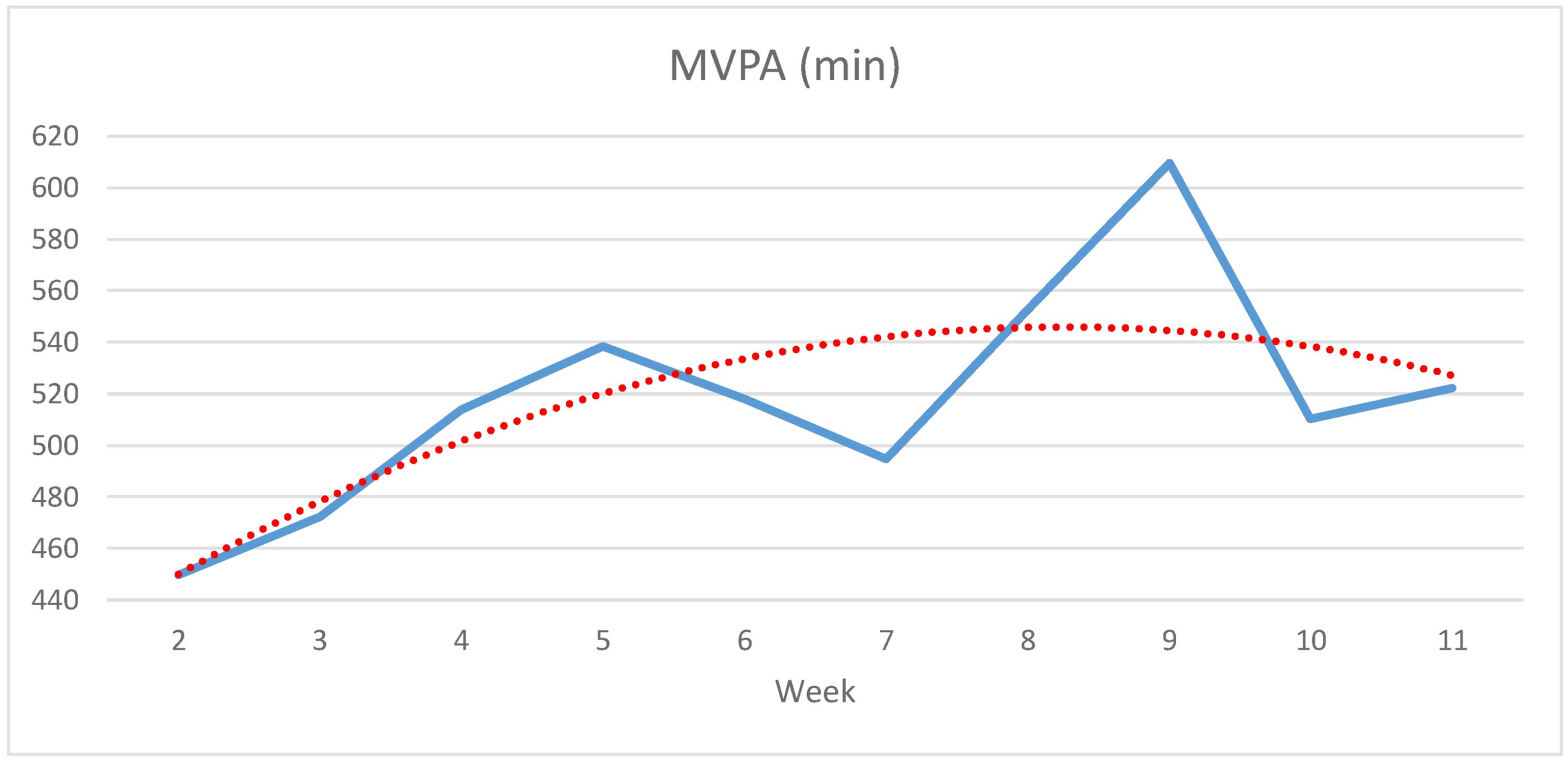
Development of average weekly MVPA in min from weeks 2 to 11 (N = 13, study group).

Fig 5 shows participants’ weekly average time of MVPA in minutes, which has a peak in both the first and second period. There is an increase of 160 min (+35.55%) between the lowest point in week 2 (450 min) and the peak in week 9 (610 min). Overall, the average increase according to the regression line is about 18% or 80 min.

### Hypothesis testing

**Fig 6:**
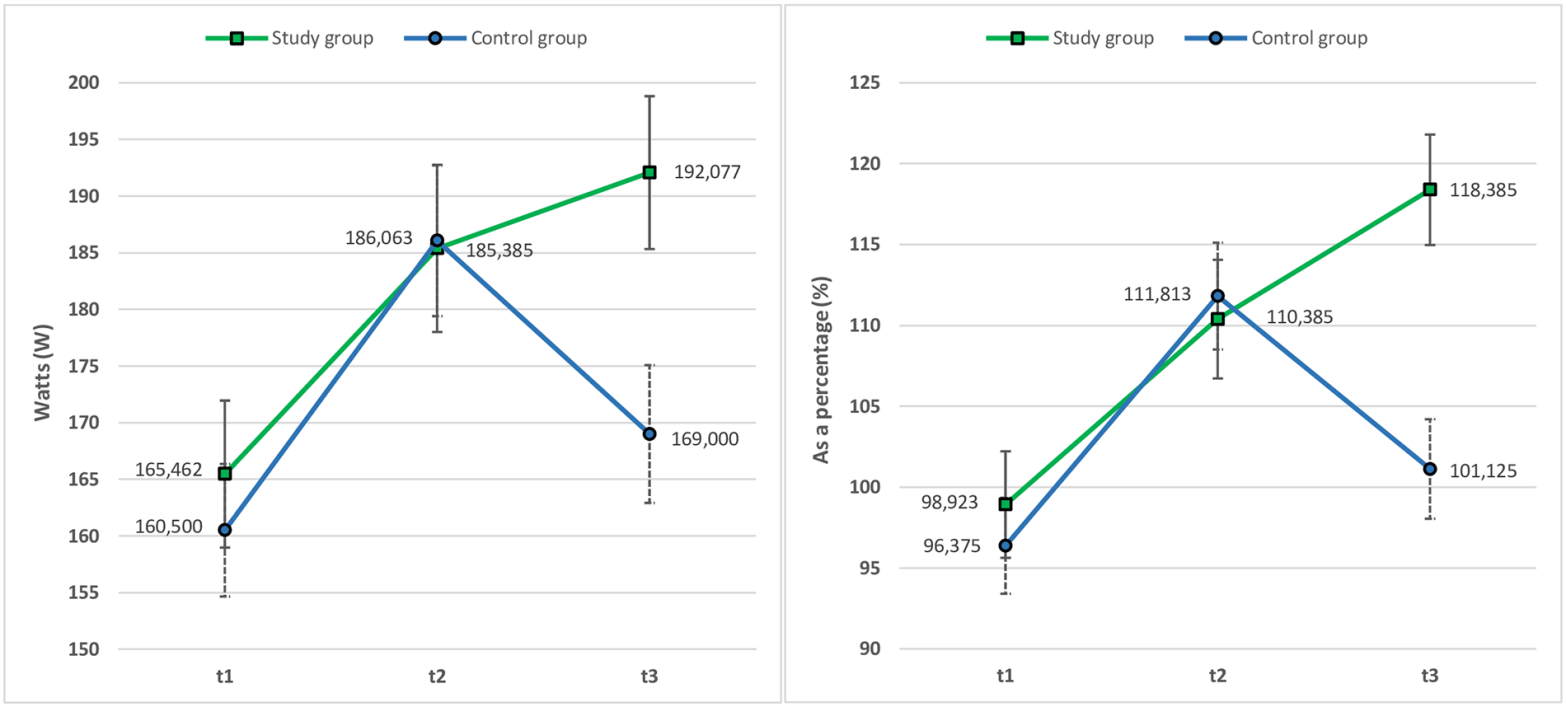
Changes in mean maximum power given in watts (left chart) and relative performance as a percentage (right chart) of study and control group at t1, t2 and t3 (examinations). Error bars represent the standard error of mean (SEM).

For data analysis, the baseline examination is referred to as *t*1, the midterm examination after 6 weeks as *t*2, and the examination after 12 weeks as *t*3. To determine whether there is a difference between the study and control groups in physiological performance, at *t*1 their mean maximum power given in W and mean relative performance as a percentage was calculated. There is no statistically significant difference between the groups in baseline maximum power (study group/control group: *M* = 165.462; SD = 51.164/*M* = 160.500; SD = 42.991; *p* = .779) and relative performance (*M* = 98.923; SD = 28.713/*M* = 96.375; SD = 18.822; *p* = .776) at *t*1. Maximum power and relative performance are then analysed by using a 2 (between-subjects factor = intervention type: being a member of either the study group or the control group) x 3 (*t*1/*t*2/*t*3) repeated measures ANOVA to find out if maximum power or relative performance differ depending on the intervention type and time of measurement. The analysis revealed a significant main effect of time for maximum power (*F*_(2,27)_ = 34.969; *p* = .000; *partial η*^*2*^ = .564) and relative performance (*F*_(2,27)_ = 23.878; *p* = .000; *partial η*^*2*^ = .469). The focus of the results especially is on the different development in maximum power between *t*2 (study group/control group: *M* = 185.385; SD = 55.206/*M* = 186.063; SD = 51.604) and *t*3 (study group/control group: *M* = 192.077; SD = 53.877/*M* = 169.000; SD = 44.077) and relative performance between *t*2 (study group/control group: *M* = 110.385; SD = 30.129/*M* = 111.813; SD = 23.092) and *t*3 (study group/control group: *M* = 118.385; SD = 27.705/*M* = 101.125; SD = 21.841) (see Fig 6). This means an improvement in maximum power of 6.69 W (+3.61%) for the study group and a deterioration of 17.06 W (-9.17%) for the control group or an improvement in relative performance of 7.99% (+7.24%) for the study group and a deterioration of 10.78 (-9.64%) for the control group. Considering the entire observation period (*t*3-*t*1), the study group improves maximum power by an average of 26.62 W (+16.09%), the control group by an average of 8.50 W (+5.30%), and the study group improves relative performance by an average of 19.49% (+19.72%), the control group by an average of 4.71% (+4.90%).

Maximum power and relative performance are relevant and most common when comparing indicators for improvement of the cardiovascular system. Indeed, there are significant differences between the study and control group according to these parameters considering the entire observation period and, in particular, between *t*2 and *t*3. Therefore, the hypothesis is accepted.

## Discussion

A strong relationship between physical activity and health has been reported in the literature. Physical activity is essential in the prevention and treatment of chronic diseases like CVD. Further, there are scientific recommendations for minimum activity levels. They show which behaviour influences the risk for chronic diseases such as CVD (e.g., [1,4,6]. Prior studies have noted the potential of smart wearables for increasing physical activity or changing behaviour towards a healthier lifestyle (e.g., [16,24,25,30]). However, no scientific study, to the best of our knowledge, is available which investigates the relationship between digital consumer wearables and health outcomes during rehabilitation in CVD patients. In addition, little insight is available on the effects of smart wearables, such as activity trackers, on behaviour change. As we know that LTPA is a factor of success in cardiac rehabilitation, the present study investigated patients who monitored their own activities and made decisive changes in activity and sedentary behaviour. It was designed to determine the effects of wearing activity tracking devices on health outcomes. Therefore, the main question in this research was whether the use of digital self-tracking for monitoring LTPA by CVD patients undergoing cardiac rehabilitation can lead to an improvement of performance of the cardiovascular system. Further, the study aimed to investigate physical activity in CVD patients and how physical activity develops throughout rehabilitation and afterwards. It was hypothesised that patients who objectively record their LTPA by using digital activity trackers show greater improvement of cardiovascular system parameters after 12 weeks, as compared to a control group without any digital activity tracking.

Over a total time period of eight calendar months, 36 CVD patients aged 44–77 were recruited to participate in a field experiment. A total of 1,059 valid days of activity data generated by 13 participants finishing the study were analysed in detail. Results provide the first complete activity profile of CVD patients on different intensity levels over a longer period of time. Weekly results were provided as an average according to number of steps, kcals burned, total activity time, sedentary behaviour and physical activity of light, moderate and vigorous intensity. Analysis of mean development of activity throughout the entire observation period supports previous research into the importance of attending rehabilitation programmes for CVD patients. They increased their weekly step-counts by almost a third throughout the study and showed an almost 10% higher energy expenditure compared to the beginning of their rehabilitation. The total activity time improved by more than 7 hours per week and sedentary behaviour was reduced by more than 6 hours each. The mean physical activity of light intensity showed an improvement of more than 20% from about 34 h to almost 40 h per week. MVPA increased by about 18% or 80 minutes. Results show varying characteristics of some activities throughout the study, especially differences between the first (weeks 1–6) and second (weeks 7–12) time period of the study. Although intensive care and supervision only took place in the first period, which was equivalent to the time period of the routine phase II cardiac rehabilitation programme, steps, total activity time, physical activities of light intensity as well as MVPA partly increased in the period following the completion of the rehabilitation programme. Further, sedentary behaviour, which remained almost constant in the first 6 weeks, was reduced by more than 4 h per week in subsequent weeks (weeks 7–12). A possible explanation for this might be that participants more or less reduced heart controlled physical exercise sessions per week in the second period and may have tried to compensate for the lack of training with a higher level of overall physical activities of lower intensity. Further, an increase of total activity time is usually accompanied with shorter periods of sitting (improvement of sedentary behaviour). In addition, weekly activity data shows changes in the middle of both periods and in the weeks following examinations. This may depend on examination results combined with medical consultation by a physician.

However, activity profiles based on objectively generated data were only created for the participants of the study group wearing an activity tracker. To compare development of performance between this group and the control group consisting of participants without any digital records, we considered examination data at three different intervals: at the beginning (*t*1), 6 weeks later at the end of phase II rehabilitation programme (*t*2), and at the very end of the 12-week study (*t*3). The focus was for the most part on physical performance data generated during cardiac stress tests including ECG and lactate measurement. Results show significant differences between the study group and control group in development of two indicators for comparing improvement or deterioration of the cardiovascular system: maximum power achieved by participants during a cardiac stress test in W and the calculated relative performance as a percentage representing the actual performance in relation to the target performance. Between *t*1 and *t*2, the development was similar for both groups. Afterwards, however, between *t*2 and *t*3, results show significant differences between the groups. Although development was not as noticeable as in the first period, the study group, at least, further improved maximum power by 3.61% and relative performance by 7.24% until *t*3. In contrast, maximum power achieved by the control group deteriorated during the same period by 9.17% and relative performance deteriorated by 9.64%. These results are astonishing as they show that maximum power achieved by participants of the control group and relative performance of this group are just slightly above baseline levels during the examination at the end of the study (*t*3) and show a significant decline in health outcomes and performance of the cardiovascular system compared to the examination at the end of the routine rehabilitation programme (*t*2).

Importantly, study group participants not only maintained the same performance level as at *t*2, they improved performance even further during the six weeks that followed. This result may be explained in several ways. One might be the fact that during the rehabilitation programme (equivalent to the first six weeks of the study) participants of both groups received intensive medical counselling, performed physical exercises under supervision and attended mandatory lectures and seminars. Afterwards, in the six weeks following *t*2, control group participants did not receive any further feedback, which may have decreased personal motivation for physical activity once they no longer received assistance from a medical team. Yet in the study group, activity trackers still provided information about daily activity and activities with different intensity levels. They provided motivating feedback, thereby encouraging participants to remain motivated or to further improve activity and sedentary behaviour leading towards a healthier lifestyle.

Even if we consider the total observation period of 12 weeks, during which both groups at least improved overall performance, we still have significant differences between the groups in development of performance. Specifically, the study group showed a more than 10% higher overall improvement of maximum power than the control group and an almost 15% higher overall improvement of relative performance. A combination of activities and diet that are supported by the tracking device and the web service for monitoring as well as feedback of the monitoring architecture has positively affected the study groups’ lifestyle. Thus, the results of the field experiment are encouraging, and provide support for the fact that smart wearables have the potential to improve the health status of patients.

### Limitations

The fact that no activity data was generated or recorded by the control group was part of the study design. Thus, the setup used in this field experiment was to compare groups by performance parameters. Therefore, physical activity or intensity levels were compared by indicators permitting conclusions to be drawn about physical activities. The sample size of both groups is comparable to other studies on activity trials over a period of 12 weeks (see, for example, [30,74]. Yet, we make a call for replication studies to further substantiate, or eventually revise, the findings of the present study. Longer periods of monitoring may provide an additional insight into development of activities or improvement of performance parameters. Further, results may not be generalizable for patients with CVD who do not undergo cardiac rehabilitation. In addition, results are only representative for patients undergoing cardiac rehabilitation at an outpatient rehabilitation centre and hence are not in a stationary rehabilitation setting. Further, caution must be exercised, as the findings of the field experiment might not be transferable to all cardiac patients because of the variety of possible disease symptoms, patterns and medical histories. Personal anamneses (e.g., blood pressure, diabetes or smoking status) were not particularly considered during the recruiting process.

### Concluding comment

The activity data generated by participants within the field experiment provides novel insights into patients’ activity, exercise and sedentary behaviour. Nearly round-the-clock monitoring was performed over a total time period of 19 weeks and an individual observation period of 12 weeks per cardiac patient. Immediate and informative feedback provided by activity trackers obviously increases self-efficacy for physical activity in CVD patients. Our results provide evidence for potentially positive effects of digital self-tracking for monitoring LTPA by CVD patients undergoing cardiac rehabilitation on performance of the cardiovascular system and improvement of physical activities.

Overall, our results are consistent with findings in the scientific literature in which authors underline the great potential of smart wearables in the management of chronic diseases and predict a promising future for them enhancing recovery in rehabilitation (e.g., [16,18,21,29]). This also has an impact on the design of self-management approaches in the rehabilitation setting. Hence, the use of smart wearables can prolong the success of the rehabilitation outside of the organized rehabilitation setting.

## Supporting information

S1 Data Figures data. This file contains the data that is behind Figs 1–5.
(XLSX)

S2 Data Figure data. This file contains the data that is behind Fig 6.
(SAV)
